# Roles of pigment epithelium-derived factor in exercise-induced suppression of senescence and its impact on lung pathology in mice

**DOI:** 10.18632/aging.205976

**Published:** 2024-06-26

**Authors:** Hiromichi Tsushima, Hirobumi Tada, Azusa Asai, Mikako Hirose, Tohru Hosoyama, Atsushi Watanabe, Taro Murakami, Masataka Sugimoto

**Affiliations:** 1 Laboratory of Molecular and Cellular Aging, Tokyo Metropolitan Institute for Geriatrics and Gerontology, Tokyo 173-0015, Japan; 2 Department of Nutrition, Shigakkan University, Aichi 474-8651, Japan; 3 Research Institute, National Center for Geriatrics and Gerontology, Aichi 474-8511, Japan

**Keywords:** senescence, exercise, PEDF, myokine, COPD

## Abstract

Senescent cells contribute to tissue aging and underlie the pathology of chronic diseases. The benefits of eliminating senescent cells have been demonstrated in several disease models, and the efficacy of senolytic drugs is currently being tested in humans. Exercise training has been shown to reduce cellular senescence in several tissues; however, the mechanisms responsible remain unclear. We found that myocyte-derived factors significantly extended the replicative lifespan of fibroblasts, suggesting that myokines mediate the anti-senescence effects of exercise. A number of proteins within myocyte-derived factors were identified by mass spectrometry. Among these, pigment epithelium-derived factor (PEDF) exerted inhibitory effects on cellular senescence. Eight weeks of voluntary running increased *Pedf* levels in skeletal muscles and suppressed senescence markers in the lungs. The administration of PEDF reduced senescence markers in multiple tissues and attenuated the decline in respiratory function in the pulmonary emphysema mouse model. We also showed that blood levels of PEDF inversely correlated with the severity of COPD in patients. Collectively, these results strongly suggest that PEDF contributes to the beneficial effects of exercise, potentially suppressing cellular senescence and its associated pathologies.

## INTRODUCTION

Mammalian cells undergo irreversible growth arrest, known as cellular senescence, which is induced by various stresses, such as DNA damage, oxidative stress, oncogene activation, and telomere shortening [1]. Cellular senescence involves the activation of tumor suppressors, such as the p19^ARF^-p53-p21 and p16^INK4a^-pRb pathways, which collectively serve as potent mechanisms inhibiting the development of cancer [2]. However, recent studies revealed a more complex and intriguing role for cellular senescence. Senescent cells accumulate in multiple tissues during aging, and this is considered to contribute to both tissue aging and the development of chronic diseases [3–5]. The senescence-associated secretory phenotype (SASP) is crucially involved in the onset of pathological conditions, and studies that employed the semi-genetic or pharmacological ablation of senescent cells demonstrated the potential therapeutic benefits of senolysis [6, 7]. The selective targeting of senescent cells was found to restore or enhance tissue function and prevent diseases, including cancer, cardiovascular disorders, neurodegenerative conditions, and age-related pathologies [8].

Chronic obstructive pulmonary disease (COPD) is a major global health concern and ranks among the leading causes of mortality, according to the World Health Organization Global Health Estimates (https://www.who.int/data/gho/data/themes/mortality-and-global-health-estimates/). Pulmonary emphysema is a major component of COPD, which is characterized by the permanent collapse of alveolar walls. Despite its prevalence and impact, there is currently no cure for this disease. Cellular senescence was shown to be enhanced in the epithelial cells of lung tissue in patients with emphysema [9]. Semi-genetic elimination of senescent cells using ARF-DTR mice, which express the diphtheria toxin receptor under the *Arf* promoter/enhancer, or p16-3MR mice, which express the herpes simplex virus thymidine kinase under the *Ink4a* promoter/enhancer, demonstrated that senescence enhanced pulmonary inflammation, thereby exacerbating their pathologies [10–12]. The senolytic intervention significantly reduced alveolar destruction and restored pulmonary function in these mice. Moreover, ABT-263 (Navitoclax), a BH3 mimetic that inhibits anti-apoptotic B-cell lymphoma (BCL)-2 family member proteins and elicits senolytic activity [13], produced similar outcomes by decreasing senescent cells and mitigating emphysema-associated pathologies in the elastase model [10]. These findings suggest the potential of preventive and therapeutic strategies for emphysema that specifically target senescent cells. Collectively, these studies highlight the detrimental role of cellular senescence and the potential of targeting senescent cells for the prevention and treatment of emphysema.

While there is currently no cure for COPD, aerobic training has been shown to exert positive effects by alleviating dyspnea and increasing exercise tolerance in patients with moderate COPD [14, 15]. However, the mechanisms underlying the beneficial effects of exercise training in COPD remain unclear. In contrast, a systematic review highlighted the potential of chronic physical training to decrease cellular senescence in peripheral T cells in humans or various tissues in rodents [16], which may imply that a reduction in cellular senescence mediates the beneficial effects of aerobic training in patients with COPD. Nevertheless, the specific mechanisms by which exercise training affects cellular senescence in tissues have not yet been elucidated. Exercise can stimulate the secretion of myokines, which is believed to contribute to the protection against several chronic diseases, including dementia and metabolic diseases [17]. Patients with COPD often experience muscle atrophy, which can be a risk factor for mortality [18], suggesting the potential involvement of myokines in their pathologies.

In the present study, we investigated myocyte-secreted factors with the potential to suppress cellular senescence, aiming to explore their protective effects against lung disease. We identified pigment epithelium-derived factor (PEDF) as a potential mediator of the suppression of senescence in response to exercise training. The expression of *Pedf* in skeletal muscle and blood PEDF levels were elevated in mice that underwent 8 weeks of voluntary exercise. PEDF exhibited the capacity to inhibit cellular senescence in cultured fibroblasts, and its administration effectively reduced senescence markers in lung and adipose tissues, mitigating pathologies in an elastase-induced pulmonary emphysema model. Moreover, an analysis of human samples indicated that serum PEDF levels were associated with respiratory parameters in patients with COPD. Therefore, the present results strongly suggest the potential of PEDF as a myokine linking exercise training to the suppression of senescence.

## RESULTS

### Myoblast-derived factor(s) suppressed cellular senescence

**Figure 1:**
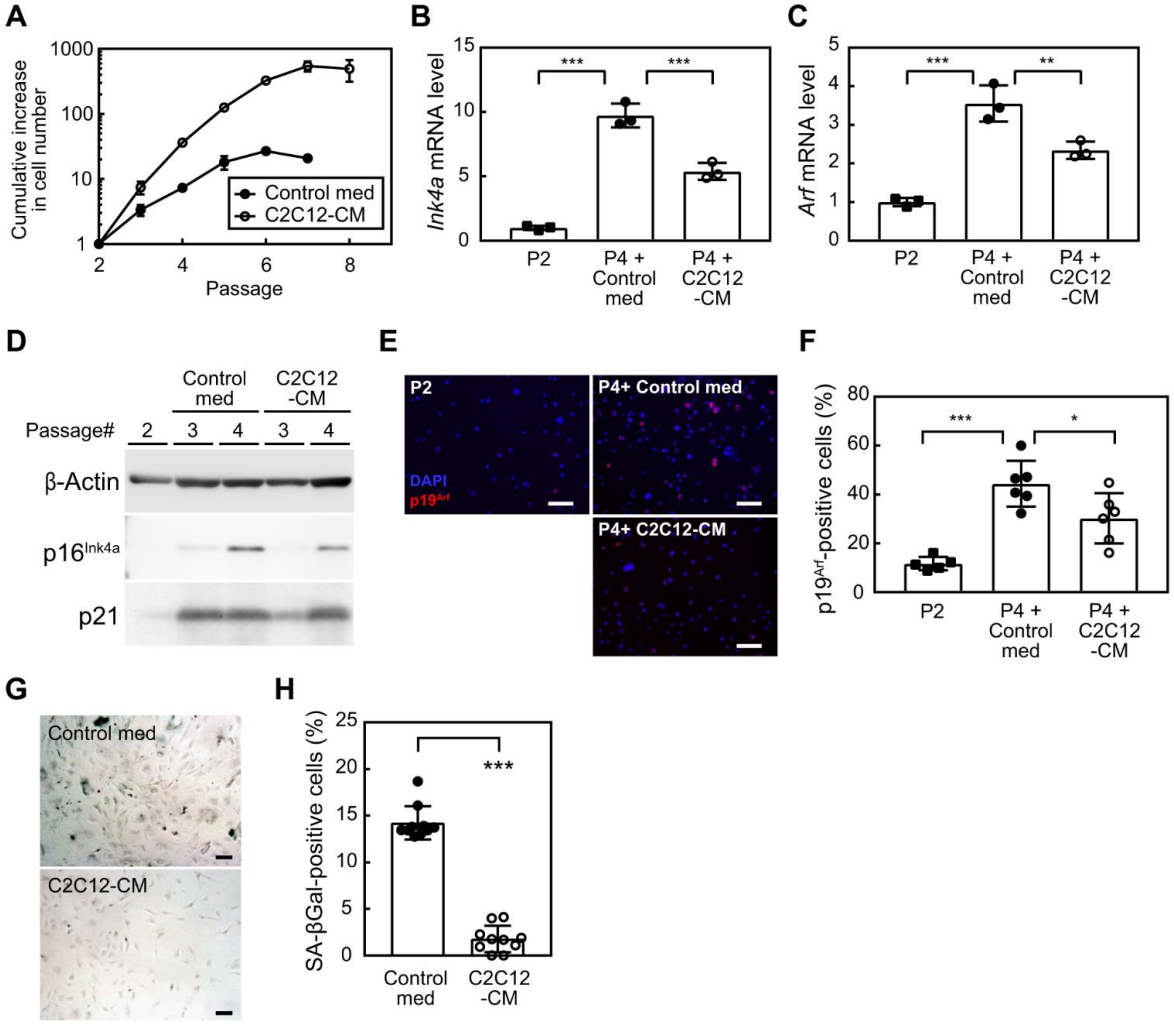
**Myocyte-derived factors suppress cellular senescence.** (**A**) Wild-type MEFs were cultured according to the 3T3 protocol in the presence of a control medium or C2C12-CM (1:1 dilution with DMEM containing 10% serum). Data represent the average value of triplicate samples ± SD. (**B**, **C**) Total RNA was isolated from cells and the expression of *Ink4a* (**B**) or *Arf* (**C**) was analyzed by real-time PCR. Values were normalized to *Gapdh* in each sample. (**D**) The expression of p16^INK4a^ and p21 was analyzed by immunoblotting. β-Actin was used as a loading control. (**E**) An immunofluorescence analysis was performed using the p19^ARF^ antibody. Cells were counterstained with DAPI. Scale bar, 100 μm. (**F**) The number of p19^ARF^-positive cells was counted in (**E**). (**G**) Cells (passage 4) were stained for SA-β-gal. Scale bar, 100 μm. (**H**) The number of SA-β-gal-positive cells was counted in each sample. Scale bar, 100 μm. Values represent means ± SD. Data were analyzed by a one-way ANOVA and Tukey’s post-hoc analysis (**B**, **C**, **F**) or the Student’s *t*-test (**H**). *P <0.05, **P <0.01, and ***P <0.001.

To investigate whether myokines play a role in mediating the exercise-induced suppression of cellular senescence, we initially examined the impact of factors derived from myoblasts on the replicative lifespan of cells. We cultured MEFs according to the 3T3 protocol [19]. MEFs were exposed to control media (control med) or C2C12-CM during the culture (1:1 dilution with 10% serum/medium). MEFs exposed to control med ceased their proliferation by passage 5 (Figure 1A). In contrast, when cultured in the presence of C2C12-CM, these cells exhibited a markedly higher rate of proliferation that persisted until passage 7. To clarify whether the senescence program was affected by C2C12-CM, we examined the expression of *Cdkn2a* (*Ink4a* and *Arf*) in these cells. The results obtained showed that the expression of both *Ink4a* and *Arf* was higher in passage 4 (P4) than in passage 2 (P2), (Figure 1B, 1C). However, MEFs exposed to C2C12-CM had lower levels of *Ink4a* and *Arf* than those exposed to control med. Consistently, cells expressed lower levels of p16^INK4a^ (Figure 1D) and a smaller number of cells expressed p19^ARF^ than control cells following exposure to C2C12-CM (Figure 1E, 1F). Additionally, p21 protein level decreased in the presence of C2C12-CM (Figure 1D). We further assessed SA-β-gal in MEFs at P4 and found that a significantly lower number of cells were positive for SA-β-gal in C2C12-exposed MEFs (Figure 1G, 1H). Collectively, these results suggest that factor(s) present in C2C12-CM possess the ability to extend the replicative lifespan of MEFs by suppressing cellular senescence.

### PEDF mediated the suppressive effect of C2C12-CM on cellular senescence

We attempted to identify the factor(s) within C2C12-CM that are responsible for suppressing cellular senescence. Proteins included in control med and C2C12-CM were separated by SDS-PAGE and visualized using silver staining (Supplementary Figure 1). We found distinct proteins in C2C12-CM in the range of 37-75 kD. These proteins were subsequently analyzed by mass spectrometry to identify the proteins in C2C12-CM. Among 841 proteins detected in C2C12-CM by mass spectrometry (Supplementary Table 1), 62 proteins were annotated as extracellular proteins (Supplementary Table 2). We further filtered candidate factors based on two criteria from the literature: 1) an increased blood concentration in response to exercise and 2) the potential to modify stress signals or cellular senescence. The selection of proteins identified by mass spectrometry based on these criteria resulted in the selection of PEDF (also known as Serpinf1 or Epc-1) as a candidate protein. Previous studies demonstrated that the expression of PEDF was induced during the myotube differentiation of human myoblasts [20] and its concentration increased in response to persistent training or exercise in both humans and mice [21, 22]. Moreover, PEDF has been shown to delay cellular senescence by reducing oxidative stress in human mesenchymal stem cells [23], and its deletion accelerated senescence in the retinal pigment epithelium of mice [24]. In the present study, we observed a significant decrease in *Pedf* expression in both the tibialis anterior (TA) and soleus (SOL) muscles of aged animals (Supplementary Figure 2). Therefore, PEDF was subjected to further analyses in the context of senescence and myokines in C2C12-CM.

**Figure 2:**
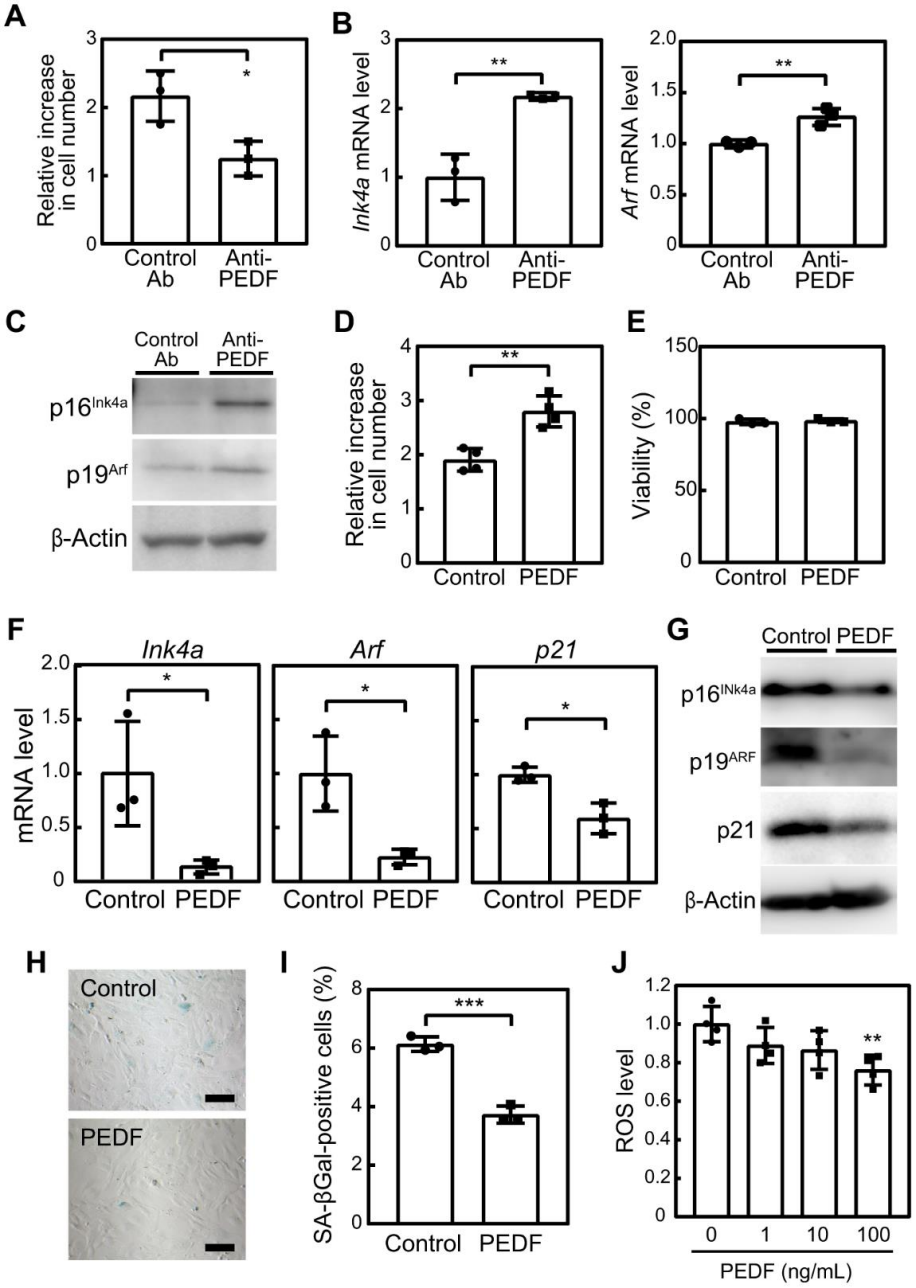
**PEDF mediates the anti-cellular senescence effects of C2C12-CM.** (**A**–**C**) MEFs were cultured in the presence of C2C12-CM treated with a control or PEDF antibody for 3 days. (**A**) Cell numbers were counted and relative changes in cell numbers in 3 days were plotted. (**B**) Total RNA was isolated from MEFs and the expression of *Ink4a* and *Arf* was analyzed by real-time PCR. Values were normalized to *Gapdh* in each sample. (**C**) p16^INK4a^ and p19^ARF^ levels were analyzed by immunoblotting. β-Actin was used as the loading control. (**D**) MEFs were cultured in the presence of a recombinant of PEDF (100 ng/mL) for 3 days. Changes in cell numbers were plotted. (**E**) Cell viability was determined by the trypan blue exclusion assay. (**F**) The expression of *Ink4a*, *Arf*, and *p21* was analyzed by real-time PCR. Values were normalized to *Gapdh* in each sample. (**G**) p16^INK4a^, p19^ARF^, and p21 levels were analyzed by immunoblotting. β-Actin was used as a loading control. (**H**) Cells were stained for SA-β-gal. Scale bar, 100 μm. (**I**) The percentage of SA-β-gal-positive cells was plotted. (**J**) Cells were stimulated with the indicated concentrations of recombinant PEDF for 3 days. Intracellular ROS levels were analyzed in each sample, and relative values were plotted against the average of the control sample. Values represent means ± SD. Data were analyzed by the Student’s *t*-test (**A**, **B**, **D**–**F**, **I**) or a one-way ANOVA and Tukey’s post-hoc analysis (**J**). *P <0.05, **P <0.01, and ***P <0.001.

To investigate the potential role of PEDF in the suppression of cellular senescence, MEFs at P3 were cultured in the presence of C2C12-CM pre-treated with either a control or neutralizing antibody against PEDF. After 3 days, we observed an increase in the population of MEFs with the control antibody (Figure 2A). The addition of anti-PEDF significantly inhibited cell proliferation. Additionally, anti-PEDF increased the expression of *Cdkn2a* (*Ink4a* and *Arf*) and the encoded proteins, p16^INK4a^ and p19^ARF^ (Figure 2B, 2C).

To further validate the effects of PEDF on cellular senescence, we investigated whether treatment with recombinant PEDF effectively inhibited senescence in MEFs. After 3 days, an increase in cell numbers was observed in PEDF-treated samples without any noticeable change in cell viability (Figure 2D, 2E). PEDF also significantly suppressed the expression of *Ink4a*, *Arf*, and *p21* as well as their corresponding proteins (Figure 2F, 2G) and reduced the number of SA-β-gal-positive cells (Figure 2H, 2I). These results strongly support the crucial role of PEDF in mediating the anti-cellular senescence effect of C2C12-CM.

We then examined PEDF signaling pathways in MEFs. Although the detailed molecular mechanisms mediating the PEDF signal remain unclear, it has been observed to lead to the activation of AKT, ERK, and p38, while down-regulating the Wnt/β-catenin signal [25, 26]. Additionally, PEDF has been reported to bind to and down-regulate the expression of the ATGL protein by promoting its ubiquitin/proteasome-dependent degradation [27]. However, in the present study, the PEDF treatment did not affect the phosphorylation of AKT, ERK, or p38 or the protein level of β-catenin or ATGL in MEFs (Supplementary Figure 3). Apart from its effects on these signaling pathways, PEDF has been shown to reduce oxidative stress, which is considered to contribute to the PEDF-mediated suppression of cellular senescence [23]. In our experiments, we observed a dose-dependent reduction in intracellular ROS levels (Figure 2J), suggesting that PEDF extended the replicative lifespan of MEFs by mitigating oxidative stress.

### Exercise stimulated PEDF expression while concurrently reducing cellular senescence

**Figure 3:**
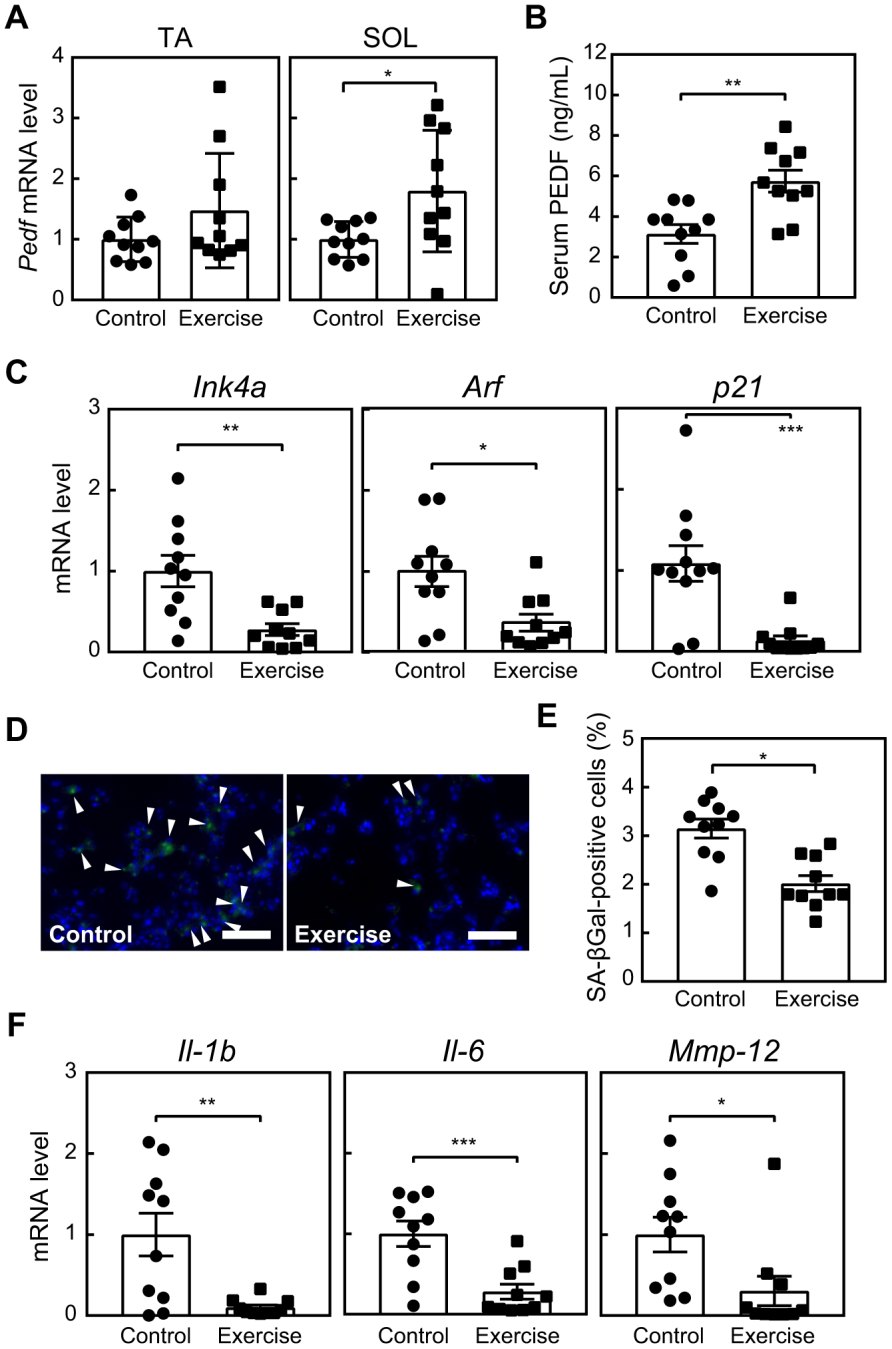
**Voluntary exercise up-regulates PEDF and suppresses cellular senescence.** Six-month-old wild-type mice were subjected to voluntary exercise for 8 weeks. (**A**) Total RNA was isolated from skeletal muscles (TA and SOL) and *Pedf* levels were analyzed by real-time PCR. Values were normalized to 18S rRNA in each sample. (**B**) Serum PEDF levels were analyzed by ELISA. (**C**) The expression of *Ink4a*, *Arf* and *p21* in the lungs was analyzed by real-time PCR. Values were normalized to *Gapdh* in each sample. (**D**) Lung sections were stained for SA-β-gal. Arrowheads indicate SA-β-gal-positive cells. Scale bar, 100 μm. (**E**) The number of SA-β-gal-positive cells was counted in each sample. (**F**) The expression of *Il-1b*, *Il-6*, and *Mmp-12* in lung total RNA was analyzed by real-time PCR. Values were normalized to 18S-rRNA in each sample. Values represent means ± SEM. Data were analyzed by the Student’s *t*-test. *P <0.05, **P <0.01, and ***P <0.001.

Due to the potential of PEDF as a myokine and its ability to delay cellular senescence *in vitro*, we were prompted to investigate its effects in a mouse exercise model. Since senescent cells become detectable in lung tissues as early as 6 months old [28], and their elimination has been shown to induce protective effects in lung disease models [10, 11], we conducted an 8-week voluntary wheel running regimen for 6-month-old mice. Control mice were maintained in cages without a running wheel. During this regimen, mice maintained an average daily running distance of more than 2 km without a significant change in their body weights (Supplementary Figure 4A, 4B). The expression of *Pedf* was increased in TA and SOL (Figure 3A), and serum PEDF levels were also elevated in the exercise group (Figure 3B). These results are consistent with previous findings indicating the increased expression of PEDF in human muscle and elevated serum PEDF levels in mice in response to training [21, 22].

Habitual physical activity and long-term exercise training have been shown to diminish the expression of p16^INK4a^ in T lymphocytes among humans, while voluntary exercise has been associated with a reduction in senescence markers, including *Cdkn2a* and SA-β-gal, across various tissues, including the heart, liver, muscles, kidneys, and fat, in mice [16]. We also observed that the expression of senescence markers, including *Ink4a*, *Arf*, *p21*, and SA-β-gal, in lung tissues was lower in the exercise group than in the control group (Figure 3C–3E). Similarly, the expression of SASP-related genes, such as *Interleukin* (*Il)-1b*, *Il-6*, and *Matrix metalloproteinase* (*Mmp*)*-12* [28], in the lungs was significantly decreased in the exercise group (Figure 3F). These results strongly suggest the potential of exercise training to reduce cellular senescence in the lungs. Although a slight change was noted in the cell composition of bronchoalveolar lavage fluid (BALF, Supplementary Figure 4C–4F), we did not observe a difference in pulmonary function between the groups (Supplementary Figure 4G–4I). Therefore, the present results were consistent with our previous findings showing that the genetic elimination of lung senescent cells at this age did not affect lung function [10].

### The administration of PEDF reduced cellular senescence in tissues

**Figure 4:**
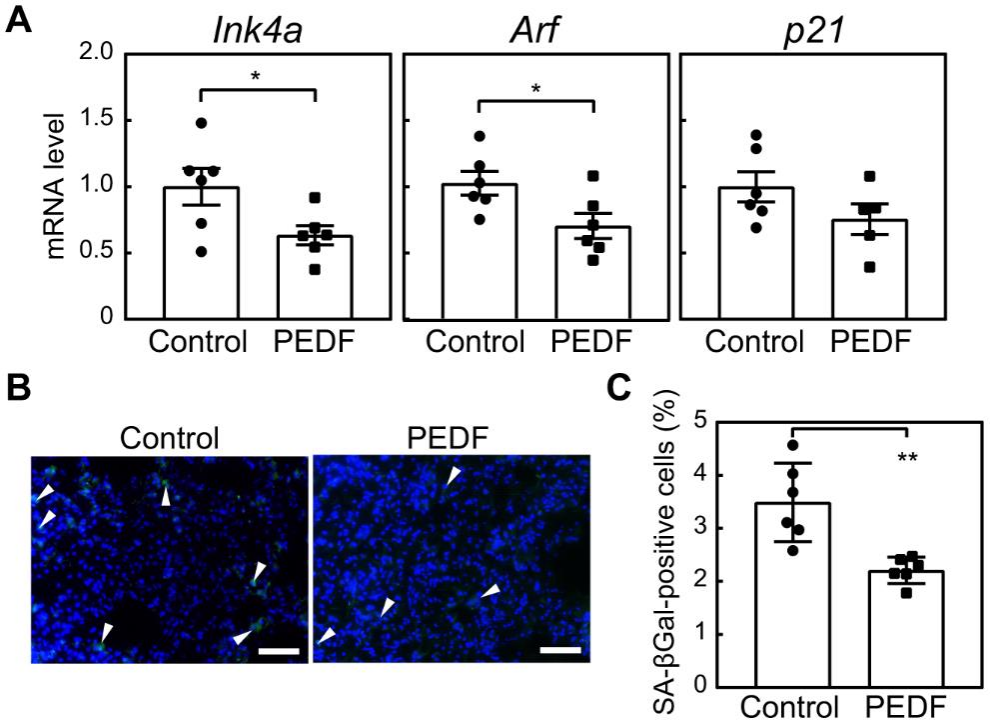
**PEDF suppresses cellular senescence in lung tissues.** A recombinant PEDF protein (10 μg/kg body weight) was intraperitoneally injected twice a week for 4 weeks. (**A**) The expression of *Ink4a*, *Arf*, and *p21* in lung total RNA was analyzed by real-time PCR. Values were normalized to *Gapdh* in each sample. (**B**) Lung tissues were stained for SA-β-gal. Arrowheads indicate SA-β-gal-positive cells. Scale bar, 50 μm. (**C**) The number of SA-β-gal-positive cells was counted in each sample. Values represent means ± SEM. Data were analyzed by the Student’s *t*-test. *P <0.05 and **P <0.01.

We investigated the relationship between elevated PEDF levels and the observed reduction in cellular senescence following exercise training. Mice were administered a recombinant PEDF protein for 4 weeks. Notably, *Ink4a*, *Arf*, and *p21* in lung tissues were decreased in the PEDF-treated group (Figure 4A). We also observed a marked reduction in the number of SA-β-gal-positive cells in lung tissues in the PEDF-treated group (Figure 4B, 4C). We then expanded our analysis to other tissues. *Ink4a*, *Arf*, and *p21* were decreased in adipose tissue in the PEDF-treated group, (Supplementary Figure 5A), and SA-β-gal activity was significantly reduced following the PEDF treatment (Supplementary Figure 5B).

To gain further insights into the role of PEDF in suppressing senescence during exercise, we examined the expression of the *Il-1b*, *Il-6*, and *Mmp-12* genes. While the expression of all of these genes in lung tissue decreased in response to exercise (Figure 3F), we specifically noted the significant down-regulation of *Mmp-12* expression in both lung and fat tissues of PEDF-treated animals (Supplementary Figure 6). Collectively, these results strongly suggest the ability of PEDF to reduce cellular senescence in multiple tissues.

### PEDF ameliorated the pathology of emphysema in mice

**Figure 5:**
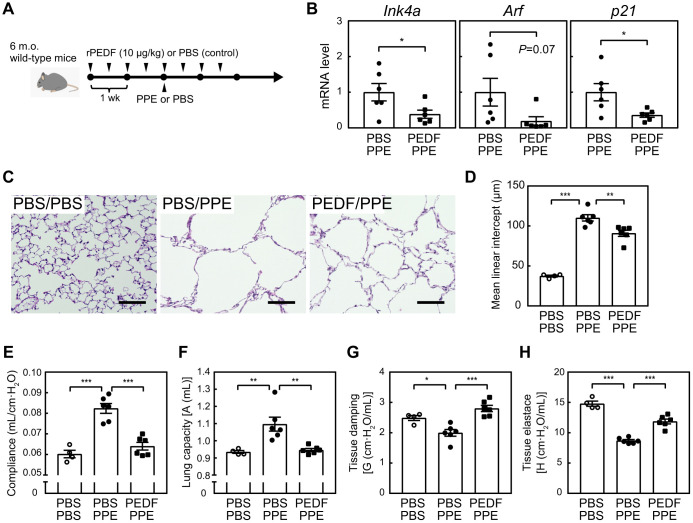
**PEDF protected lung tissues from PPE-induced emphysema.** (**A**) Experimental design. A recombinant PEDF protein was injected intraperitoneally twice a week for 4 weeks. PPE (5 units) was intranasally administered 2 weeks after the first dose of PEDF. Mice were analyzed 3 weeks after the PPE treatment. (**B**) Total RNA was isolated from lung tissues and the expression of *Ink4a*, *Arf*, and *p21* was analyzed by real-time PCR. Values were normalized to *Gapdh* in each sample. (**C**) Representative images of control and PEDF-treated mouse lung sections. Sections were stained with hematoxylin and eosin. Scale bar, 100 μm. (**D**) Alveolar mean linear intercepts were measured. (**E**–**H**) A pulmonary function test was performed. Lung compliance (**E**), capacity (**F**), tissue damping (**G**), and tissue elastance (**H**) are shown. Values represent means ± SEM. Data were analyzed by the Student’s *t*-test (**B**), or a one-way ANOVA and Tukey’s post-hoc analysis (**D**–**H**). *P <0.05, **P <0.01, and ***P <0.001.

We previously demonstrated that the semi-genetic or pharmacological elimination of senescent cells in the lungs protected against pulmonary emphysema induced by elastase or cigarette smoking in 6-month-old mice [10, 11]. Based on the ability of PEDF to suppress cellular senescence in lung tissue in mice, we investigated whether it also exerted protective effects in a mouse emphysema model. Mice were pre-administered with a recombinant PEDF protein and were then subjected to an elastase (PPE) treatment in order to induce pulmonary emphysema, as shown in Figure 5A. While PPE does not affect the *Ink4a* and *Arf* levels in the lung tissues [10], senescence markers were decreased in the lung tissues of the PEDF-treated group, indicating that cellular senescence was also diminished following the PEDF treatment in the elastase-induced emphysema model (Figure 5B). A morphometric analysis of inflated lung sections revealed that PPE induced a massive alveolar collapse (compare PBS/PBS and PBS/PPE in Figure 5C, 5D), which was diminished by pre-administration of PEDF, suggesting that PEDF suppressed PPE-induced alveolar collapse. Consistently, a spirometric analysis demonstrated that the PEDF treatment significantly protected against PPE-induced pulmonary dysfunction (Figure 5E–5H). The administration of PEDF did not affect the cell composition of BALF in the PPE-induced emphysema model (Supplementary Figure 7), which was consistent with our previous findings showing that PPE only induced the transient accumulation of inflammatory cells following its installation [10]. Collectively, these results suggest that the PEDF treatment effectively protects against pulmonary emphysema by inhibiting cellular senescence.

To clarify the relevance of PEDF in human pathology, we investigated whether serum PEDF levels correlated with respiratory function in patients diagnosed with COPD (Supplementary Table 3). Serum PEDF levels in 20 patients were measured by ELISA and respiratory function parameters were assessed by the forced expiratory volume in 1 second per forced vital capacity of the lung (FEV1/FVC). A weak correlation was observed between serum PEDF level and respiratory function through linear regression analysis (r=0.48 and *P*=0.03, Supplementary Figure 8), suggesting a potential involvement of PEDF in the pathology of COPD in humans.

### Potential of miR-127 as a mediator of the PEDF-induced inhibition of cellular senescence

**Figure 6:**
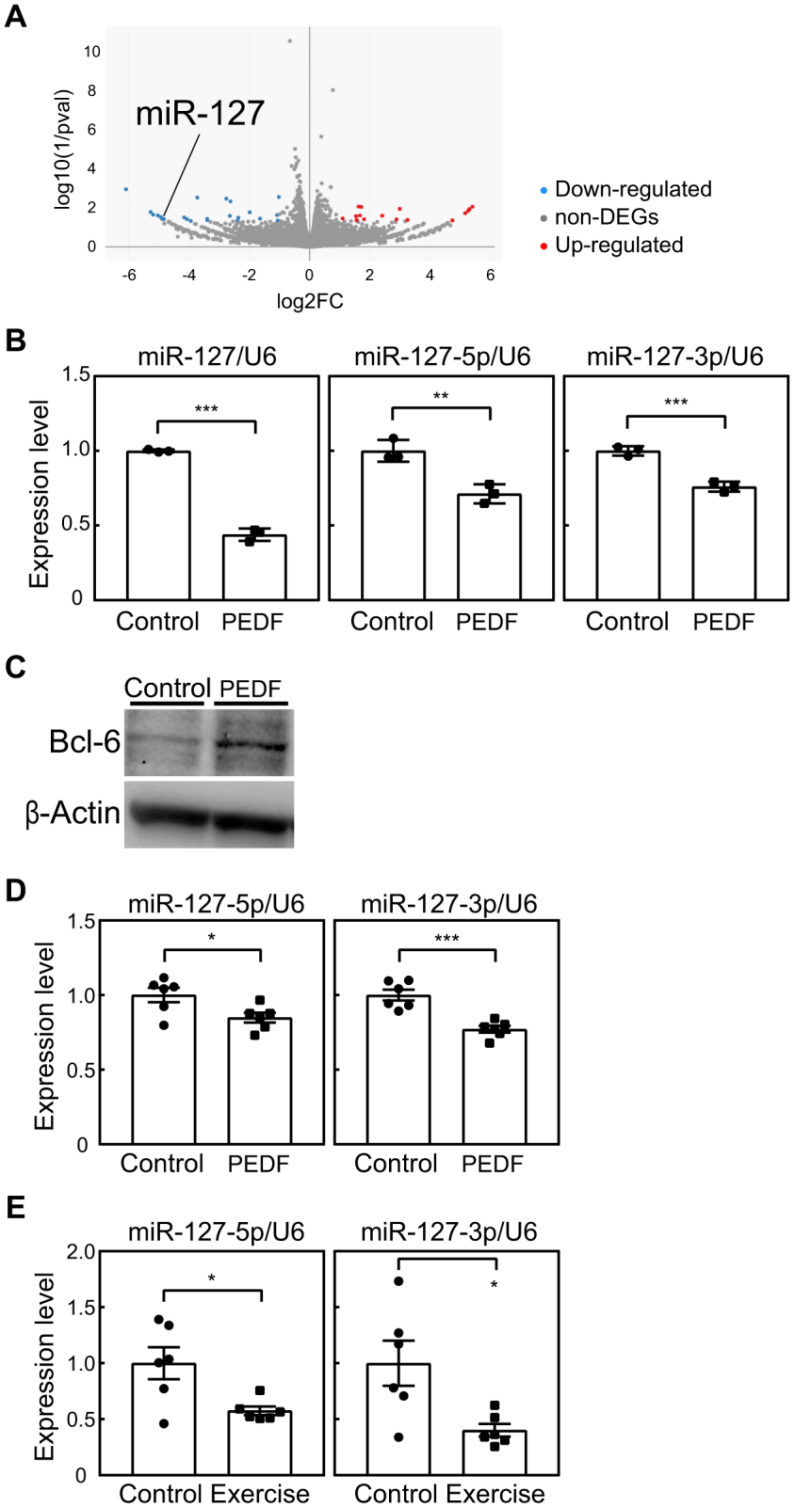
**Involvement of miR-127 in PEDF signaling.** (**A**) An RNA sequencing analysis was performed on MEFs stimulated with a recombinant PEDF protein. A volcano plot of RNA sequencing data is shown. A full list of RNA sequencing data is available in the Gene Expression Omnibus database (accession number; GSE241459). (**B**) The expression of miR-127 (-loop, -5p, and -3p) in MEFs was analyzed by real-time PCR. Values were normalized to U6 snRNA in each sample. (**C**) The expression of BCL-6 in MEFs was analyzed by immunoblotting. β-Actin was used as a loading control. (**D**, **E**) miR-127 levels in the lungs of mice subjected to the PEDF treatment (**D**, Figure 4) or voluntary wheel running (**E**, Figure 3) were analyzed by real-time PCR. miR-127 levels were normalized to U6 snRNA in each sample. Values represent means ± SD (**B**) or means ± SEM (**D**, **E**). Data were analyzed by the Student’s *t*-test. *P <0.05, **P <0.01, and ***P <0.001.

The results presented above strongly indicated the potential of PEDF to suppress cellular senescence, thereby mediating the beneficial effects of exercise training in mice. Although we demonstrated that PEDF reduced intracellular ROS levels in MEFs (Figure 2J), the underlying mechanisms remain unclear. Therefore, we attempted to identify factor(s) that potentially mediate the function of PEDF. We performed an RNA sequencing analysis of MEFs (P3) stimulated or not with recombinant PEDF. We identified 40 differentially expressed genes, including 16 up-regulated genes and 24 down-regulated genes, between control and PEDF-treated cells (Figure 6A and Supplementary Table 4). Of these genes, miR-127, which was down-regulated by PEDF in MEFs, was previously shown to increase during senescence and promote cellular senescence in mouse and human fibroblasts [29, 30]. The PEDF-mediated down-regulation of miR-127 in MEFs was further confirmed by real-time PCR (Figure 6B). miR-127 directly targets *BCL-6*, and its down-regulation has been shown to contribute to the induction of senescence by miR-127 [31]. Therefore, we examined BCL-6 expression levels in MEFs. BCL-6 protein expression levels were higher in PEDF-treated MEFs than in control cells (Figure 6C), suggesting a role for the miR-127-BCL-6 axis in the regulation of senescence by PEDF.

We subsequently examined miR-127 levels in the lung tissues of PEDF-treated animals. A slight decrease was observed in miR-127 levels in PEDF-treated animals, which indicated that PEDF exhibited the ability to down-regulate miR-127 *in vivo* (Figure 6D). Notably, miR-127 levels were significantly reduced in the lungs of mice subjected to voluntary exercise for 8 weeks (Figure 6E). Collectively, these results strongly suggest that PEDF is intricately linked to the anti-senescence effects of exercise through its suppression of miR-127.

## DISCUSSION

Regular physical activity plays a crucial role in promoting healthy aging and is beneficial in the management of chronic diseases, such as COPD [32, 33]. Conversely, physical inactivity contributes to the development of chronic diseases [34]. However, the specific mechanisms that mediate the effects of exercise remain unclear. Habitual physical activity has been associated with lower levels of p16^INK4a^ in the immune cells of healthy humans [35–37]. Similarly, the expression of senescence markers was reduced in the heart, vessels, endothelium, and adipose tissues of animals undergoing prolonged voluntary exercise [38–41]. Recent advances in the field of cellular senescence, particularly studies using senolysis models, have provided compelling evidence for the involvement of senescence in the pathologies of many chronic diseases [6]. Therefore, anti-senescence effects may mediate the beneficial outcomes of exercise.

We herein focused on myokines, particularly those up-regulated in response to exercise, as potential mediators of the anti-senescence effects of exercise. Several factors have been identified as myokines, with some demonstrating exerkine properties that benefit the cardiovascular, metabolic, immune, and neurological systems [42]. Among these, irisin has been shown to suppress cellular senescence [43, 44], and its blood concentration has been associated with physical activity [45]. In addition, irisin levels inversely correlated with the severity of COPD in patients [46], and were increased by exercise training in both humans and animal models [45, 47]. However, irisin was not detected in C2C12-CM, which has the potential to extend the replicative lifespan of MEFs (Figure 1), indicating that irisin is unlikely to mediate the anti-senescence effects of C2C12 cells. We identified PEDF as a potential suppressor of cellular senescence that is included in C2C12-CM. The inactivation of PEDF increased senescence markers, and its administration was sufficient to reduce senescence in MEFs (Figure 2), suggesting that PEDF mediates the anti-senescence effects of C2C12-CM.

The increase in blood PEDF has been demonstrated in the aerobic training model [22]. *Pedf* expression is enhanced in muscles following the training, which could contribute to the changes in systemic PEDF levels. While we cannot exclude the possibility of PEDF production in other tissues or the contribution of other myokines/exerkines to the suppression of cellular senescence during exercise, the administration of PEDF to mice also reduced senescence markers in the lung and fat. However, the effect of PEDF administration on SASP expression is limited (Supplementary Figure 6) compared to that of the exercise training (Figure 3F). These findings suggest that PEDF may not be the sole factor responsible for mediating senescence suppression by exercise. Other factors may collaborate with PEDF to elicit the beneficial effect of exercise training. While we used female mice in this study due to their increased susceptibility to COPD [48], our previous analysis revealed that senescent cell elimination elicits protective effects against COPD-associated pathology [10]. However, it should be carefully determined whether the production and function of PEDF vary between genders.

Although PEDF did not affect previously defined signals in MEFs, it decreased intracellular ROS levels (Figure 2), as was previously observed in human mesenchymal stem cells [23]. The specific signaling pathways connecting PEDF to ROS or the suppression of senescence remain unclear. However, our RNA sequencing analysis showed that miR-127 was down-regulated in both cultured cells and lung tissues after the administration of PEDF or voluntary exercise (Figure 6). A previous study revealed that miR-127 levels were increased in senescent fibroblasts [30]. Conversely, the ectopic expression of miR-127 has been shown to induce senescence [29, 31]. One of the targets of miR-127 is *Bcl-6* [31]. BCL-6 has been shown to prevent the increase in intracellular ROS levels induced by genotoxic agents [49], which may explain the observed reduction in ROS levels in MEFs (Figure 2J). Furthermore, the overexpression of BCL-6 has been shown to suppress cellular senescence in mouse fibroblasts [50]. A PEDF stimulation increased BCL-6 levels in MEFs, indicating a potential role for the miR-127/BCL-6 pathway in the PEDF signal. However, BCL-6 protein levels were below the detection limit in the lung tissues of mice, and other miR-127 targets or PEDF signaling pathways may also contribute to the outcome of PEDF administration or exercise training. Further studies are required to define the PEDF signal that suppresses cellular senescence.

Cellular senescence was previously shown to be accelerated in the lung tissues of patients with COPD [9]. Furthermore, the conditional ablation of senescent cells attenuated emphysema-associated pathologies in mouse models [10, 28]. The present results indicate that PEDF restored pulmonary function in an emphysema model while concurrently decreasing the number of senescent cells. This supports the hypothesis that PEDF may mediate the positive effects of exercise by suppressing senescence. Physical activity is the most significant prognostic factor for patients with COPD, and exercise therapy is commonly used to decelerate progression in these patients [33]. The severity of COPD is frequently associated with sarcopenia [51], suggesting the involvement of myokines in the pathology. Exercise training, which may restore muscle mass, could therefore be an effective approach to maintaining pulmonary function in COPD. Indeed, we discovered a weak, but important correlation between blood PEDF levels and respiratory function in COPD patients, indicating the involvement of PEDF in the pathology of COPD in humans. Additionally, blood PEDF levels in humans were much higher than those observed in mice (Supplementary Figure 8), possibly due to the disparity in muscle mass amount between the two species, which could facilitate the detection of blood PEDF in humans. Therefore, blood PEDF levels may be utilized to predict the severity or risk of emphysema.

Possible links between PEDF and neurological diseases, such as dementia and age-related macular degeneration (AMD), have been reported [52–54]. Cellular senescence has been implicated in these diseases [55, 56]. Additionally, physical activity has been associated with a decreased risk of dementia and AMD [57, 58]. These findings suggest the involvement of PEDF in suppressing cellular senescence, which may be linked to these neurological disorders. Furthermore, we observed the significant down-regulation of *Pedf* expression in the skeletal muscle of aged animals (Supplementary Figure 2). Since cellular senescence is known to contribute to frailty in aged animals [59], the decrease in PEDF levels associated with aging may contribute to senescence-induced frailty. PEDF is expressed in a wide variety of tissues, as reported in the Gene Expression Omnibus database. Future studies are needed to investigate whether PEDF levels change in other tissues during aging as well as the mechanisms by which PEDF in other tissues may affect aging and disease phenotypes.

While senolysis is promising based on its efficacy in animal models, its application to humans requires caution due to its potential adverse effects. Exercise therapy may be a safer approach to providing senolytic or seno-suppressive treatment [16]. However, some studies reported contradictory increases in senescence markers with forced exercise in animals [60, 61], suggesting that the impact of exercise on cellular senescence is affected by the type and intensity of exercise. Based on the present results, PEDF represents an alternative to controlling cellular senescence. However, the physiological activity of PEDF remains unclear. Further investigations are warranted to clarify the specific mechanisms and pathways through which PEDF may contribute to aging and disease phenotypes, offering valuable insights for potential therapeutic interventions.

## MATERIALS AND METHODS

### Animals

Six-month-old female C57BL/6J mice were purchased from Japan SLC Inc. and maintained under specific pathogen-free conditions with a 12-h light/dark cycle, constant temperature, and *ad libitum* access to food (CRF-1, Oriental Yeast Co., Ltd.) and drinking water. As the exercise model, mice were independently housed in a cage with a 15-cm running wheel with a rotation sensor (ML-321, Marukan Co., Ltd.).

Regarding the administration of PEDF, a recombinant PEDF protein (PKSM040786, Elabscience Biotechnology Inc.) diluted with phosphate-buffered saline (PBS) was intraperitoneally injected at a dose of 10 μg/kg body weight twice a week for 4 weeks.

The pulmonary emphysema model was prepared as previously described [62]. Five units of porcine pancreatic elastase (PPE) (EC134, Elastin Products Company, Inc.) in 100 μl PBS was intranasally administered using a standard pipette tip.

### Cell culture

Mouse embryonic fibroblasts (MEFs) were prepared from C57BL/6J embryos (embryonic day 13.5). MEFs were maintained in Dulbecco’s Modified Eagle Medium (DMEM, 045-30285, Fujifilm Corp.) supplemented with 10% fetal bovine serum (FBS), non-essential amino acids (11140050, Thermo Fisher Scientific Inc.), 10 μg/mL gentamicin (15710064, Thermo Fisher Scientific Inc.), and 55 μM 2-mercaptoethanol. MEFs were cultured in atmospheric O_2_ (20%) and 5% CO_2_ according to the 3T3 protocol [19]. Cell viability was evaluated by the trypan blue exclusion test.

C2C12 cells were maintained in DMEM supplemented with 10% FBS and gentamicin. To induce terminal differentiation, cells were cultivated in DMEM supplemented with 2% horse serum and Insulin-Transferrin-Selenium (41400045, Thermo Fisher Scientific Inc.) for 2 days. Conditioned media were then collected from differentiated C2C12 cells, filtered, aliquoted, and stored at -80°C until used. Conditioned media were diluted with an equal volume of MEF medium for use on MEFs. To neutralize PEDF, the conditioned media were incubated with anti-PEDF (5 μg/mL, PAB972Mu02, Cloud-Clone Corp.) at room temperature for 1 hour before use.

### Mass spectrometry

Proteins in control media and conditioned media prepared from differentiated C2C12 myoblasts (C2C12-CM) (200 μL) were analyzed by mass spectrometry. Albumin was removed using the ProMax Albumin Removal kit (24351, Polysciences Inc.). Samples were reduced with 20 mM Tris (2-carboxyethyl) phosphine at 80°C for 10 minutes, followed by alkylation with 30 mM iodoacetamide at room temperature for 30 minutes. Proteins were digested with 500 ng Trypsin/Lys-C Mix (V5071, Promega Corp.) at 37°C overnight.

Extracted peptides were then separated via nanoLC (Ultimate 3000 RSLCnano LC system, Thermo Fisher Scientific Inc.), and the LC eluent was coupled to an electrospray-ionization source attached to the Q Exactive HF-X mass spectrometer (Thermo Fisher Scientific Inc.). MS/MS spectra were analyzed using Scaffold DIA (Proteome Software, Inc.). Significance thresholds were selected based on the peptide false discovery rate (FDR) and protein FDR, and data were considered to be significant when each showed a value less than 0.01.

### Real-time PCR

Tissue samples were collected after the pulmonary function tests (described below), snap-frozen, and stored at -80°C until used. TA and SOL were collected from the hind limb, and the right lung lobes were used for the RNA analysis. Total RNA was extracted using TRI Reagent® (TR118, Molecular Research Center, Inc.) according to the manufacturer’s instructions. RNA was reverse-transcribed using the PrimeScript RT Reagent Kit with gDNA Eraser (RR047A, Takara Bio Inc.). Real-time PCR was performed on a CFX Connect Real Time System (Bio-Rad Laboratories, Inc.) using the following primers: *Arf*, 5’-GCCGCACCGGAATCCT-3’ (sense) and 5’-TTGAGCAGAAGAGCTGCTACGT-3’ (antisense); *Gapdh*, 5’-AATGGTGAAGGTCGGTGTG-3’ (sense) and 5’-GAAGATGGTGATGGG CTTCC-3’ (antisense); *Il-1b*, 5’-GAATGCCACCTTTTGACAGTG-3’ (sense) and 5’-CTGGATGC TCTCATCAGGACA-3’ (antisense); *Il-6*, 5’-TAGTCCTTCCTACCCCAATTTCC-3’ (sense) and 5’-TTGGTCCTTAGCCACTCCTTC-3’ (antisense); *Ink4a*, 5’-CCCAACGCCCCGAACT-3’ (sense) and 5’-GCAGAAGAGCTGCTACGTGAA-3’ (antisense); *p21,* 5’-CGAGAACGGTGGAACTTTGAC-3’ (sense) and 5’-CAGGGCTCAGGTAGACCTTG-3’ (antisense); *Mmp-12*, 5’-CTGCTCCCATGAATGAC AGTG-3’ (sense) and 5’-AGTTGCTTCTAGCCCAAAGAAC-3’ (antisense); *Pedf*, 5’-CCAACTTC GGCTACGATCTGT-3’ (sense) and 5’-TCTGTTCGATGTTCAGCTCCC-3’ (antisense); 18S rRNA, 5’-AGTCCCTGCCCTTTGTACACA-3’ (sense) and 5’-GATCCGAGGGCCTCACTAAAC-3’ (antisense).

MicroRNA was analyzed using the Mir-X™ miRNA qRT-PCR TB Green® Kit (Z8314N, Takara Bio Inc.). The following primers were used to detect miR-127: miR-127 loop, 5’-GGCTCTGATTCAGAAAGATC-3’; miR-127-5p, 5’-CTGAAGCTCAGAGGGCTCTGAT-3’; miR-127-3p. 5’-TCGGATCCGTCTGAGCTTGGCT-3’.

### RNA sequencing

Total RNA was extracted from MEFs cultured in the presence or absence of 100 ng/mL of PEDF for 24 hours using NucleoSpin RNA (740955, MACHEREY-NAGEL Inc.) according to the manufacturer’s instructions. The quality of RNA was assessed using an Agilent 2100 Bioanalyzer (Agilent Technologies, Inc.). mRNA was isolated from total RNA using the NEBNext® Poly(A) mRNA Magnetic Isolation Module (E7490, New England Biolabs, Inc.). Libraries were generated using the NEBNext® Ultra™II Directional RNA Library Prep Kit (E7760, New England Biolabs, Inc.) and sequenced on Illumina NovaSeq 6000 (Illumina, Inc.) at Rhelixa, Inc. Raw paired-end sequence reads were evaluated for quality using FastQC (Version 0.11.7; https://www.bioinformatics.babraham.ac.uk/projects/fastqc/) [63]. Trimmomatic software (Version 0.38) was employed to trim low-quality bases (<20) and remove adapter sequences [64]. The specific parameters used for this process were as follows: ILLUMINACLIP: path/to/adapter.fa:2:30:10, LEADING:20, TRAILING:20, SLIDINGWINDOW:4:15, MINLEN:36. Processed reads underwent alignment to the reference genome using the RNA-seq aligner HISAT2 (Version 2.1.0) [65]. The resulting .sam files from HISAT2 were then converted to the .bam format using Samtools (Version 1.9). The featureCounts tool (Version 1.6.3) was then applied to .bam files in order to estimate the abundance of uniquely mapped reads [66]. Raw read counts underwent normalization using relative log normalization, and a differential expression analysis was performed using DESeq2 (Version 1.24.0) [67]. Full data are available from the Gene Expression Omnibus database of the National Center for Biotechnology Information (accession number: GSE241459).

### Immunoblotting

Lysates were prepared using a buffer containing 10 mM Na-phosphate (pH 7.2), 150 mM NaCl, 2 mM EDTA, 0.1% SDS, 1% Na-deoxycholate, and 1% NP-40 and protease inhibitors. Lysates were separated by SDS-PAGE and transferred to PVDF membranes (Merck KGaA). Proteins were detected with antibodies to p19^ARF^, p21, BCL-6, phospho-ERK1/2 (sc-32748, sc-6246, sc-7388, and sc-136521; all from Santa Cruz Biotechnology, Inc.), β-Actin, β-catenin, phospho-Akt (ser473), phospho-p38 (#12620, #9562, #4060, and #9211; all from Cell Signaling Technology, Inc.), adipose triglyceride lipase (ATGL) (55190-1-AP, Proteintech, Inc.), and p16^INK4a^ [68]. Antibodies were diluted to 1:200 (BCL-6), 1:500 (p16^INK4a^, p19^ARF^, p21, phospho-Akt (ser473), phospho-ERK1/2, and ATGL), 1:1000 (β-catenin, phospho-p38), or 1:2000 (β-Actin) with a blocking buffer containing 5% skim milk, 0.1% Tween 20 in Tris-buffered saline (pH 7.5, TBS), or a blocking buffer containing 4% bovine serum albumin, and 0.1% Tween 20 in TBS for phospho-Akt, phospho-ERK1/2, and phospho-p38 antibodies.

### Morphometry

The lungs were inflated by injecting Mildform®20N (Fujifilm Corp.) and fixed for 10 minutes at constant pressure (25 cmH_2_O) [62]. Paraffin-embedded tissues were sectioned (thickness of 5 μm) and stained with hematoxylin and eosin. At least eight randomly selected fields per mouse were photographed. Test lines were randomly drawn on the images, and intercepts with the tissue structure were counted for each line. Airway and vascular structures were eliminated from the analysis.

### Immunofluorescence

MEFs were seeded onto coverslips and fixed with 4% paraformaldehyde for 20 minutes. Cells were permeabilized in 0.5% Triton X-100 and immunostained using p19^ARF^ (1:200 dilution, sc-32748, Santa Cruz Biotechnology, Inc.). The sites of antibody binding were visualized using Cy3-conjugated anti-rat IgG (1:200 dilution, 712-165-153, Jackson ImmunoResearch Inc.) and mounted using DAPI Fluoromount-G® (0100-20, Southern Biotechnology Associates Inc.). Fluorescence images were acquired using a fluorescence microscope (BX-X710, Keyence Corp.).

### Senescence-associated β-galactosidase (SA-β-gal) staining

Frozen 10-μm-thick lung sections were fixed with 4% paraformaldehyde for 20 minutes and stained using the Cellular Senescence Detection Kit-SPiDER-bGal (SG03, Dojindo Molecular Technologies, Inc.). Sections were mounted using DAPI Fluoromount-G®.

Adipose tissue (1-cm squares) and MEFs were stained using an SA-β-gal staining Kit (#9860, Cell Signaling Technology, Inc.). In adipose tissue staining, images in the RGB format were analyzed using ImageJ. The blue-green signal intensity per tissue area was quantified in each sample.

### ROS assay

MEFs were seeded on a 96-well plate (10,000 cells/well) and treated with PEDF for 24 hours. Intracellular ROS levels were analyzed using a ROS Assay Kit - Highly Sensitive DCFH-DA- (R252, Dojindo Molecular Technologies, Inc.) according to the manufacturer’s instructions. Fluorescence intensity was measured by a microplate reader (the VICTOR Nivo Multimode Microplate Reader, PerkinElmer Inc.).

### Pulmonary function tests

Pulmonary function tests were performed using a flexiVent system (Emka Technologies, Inc.) as previously reported [28]. Mice were euthanized by an intraperitoneal injection of pentobarbital sodium (100 mg/kg body weight) and connected to the flexiVent system. They were ventilated at a respiratory rate of 159 breaths per minute with a tidal volume of 10 mL/kg against a positive end-expiratory pressure of 3 cmH_2_O. Deep inflation, Snapshot-150, Quickprime-3, and a pressure-volume loop with a constantly increasing pressure were consecutively performed 3 times in each mouse.

### Analysis of bronchoalveolar lavage fluid (BALF) cells

BALF cells were analyzed as previously reported [10]. In brief, BALF cells were prepared with 1 mL of PBS containing 5 mM EDTA, and cells were collected from BALF by mild centrifugation. Collected cells were attached to glass slides using StatSpin Cytofuge (Beckman Coulter, Inc.) and subjected to modified Giemsa staining with a Diff-Quick stain kit (Sysmex Corp.).

### Enzyme-linked immunosorbent assay

Mouse blood samples were collected from the abdominal vena cava prior to conducting pulmonary function tests using a 24G needle (a minimum of 0.3 mL blood sample was obtained from each mouse). Samples were analyzed using a Mouse PEDF, PEDF ELISA Kit (CSB-E08820m, Cusabio Technology, LLC). Human sera (information including age, sex, and pathology are listed in Supplementary Table 3) were purchased from ProteoGenex Inc. and PEDF levels were analyzed using a Human PEDF ELISA kit (RD191114200R, BioVender R&D).

### Statistical analysis

A two-tailed unpaired Student’s *t*-test was used to compare two sets of experimental data. A one-way ANOVA was performed to compare more than two sets of data. When the statistical model was proven to be significant, differences between combinations of the two groups were analyzed using the Tukey-Kramer test. Data were analyzed by GraphPad Prism 7.0 (GraphPad Software Inc.). Significance was represented by asterisks as follows: *P <0.05, ** *P* <0.01, and *** *P* <0.001. No statistical method was used to select the sample size.

## Supplementary Material

Supplementary Figures

Supplementary Table 1

Supplementary Table 2

Supplementary Table 3

Supplementary Table 4
