# Supplementary material for: Roles of pigment epithelium-derived factor in exercise-induced suppression of senescence and its impact on lung pathology in mice
Source: Aging (Albany NY). 2024 Jun 26;16(13):10670–93. doi: 10.18632/aging.205976 (PMC11272117; doi:10.18632/aging.205976)
Supplement: Supplementary Figures [file aging-16-205976-s001.pdf]

SUPPLEMENTARY FIGURES

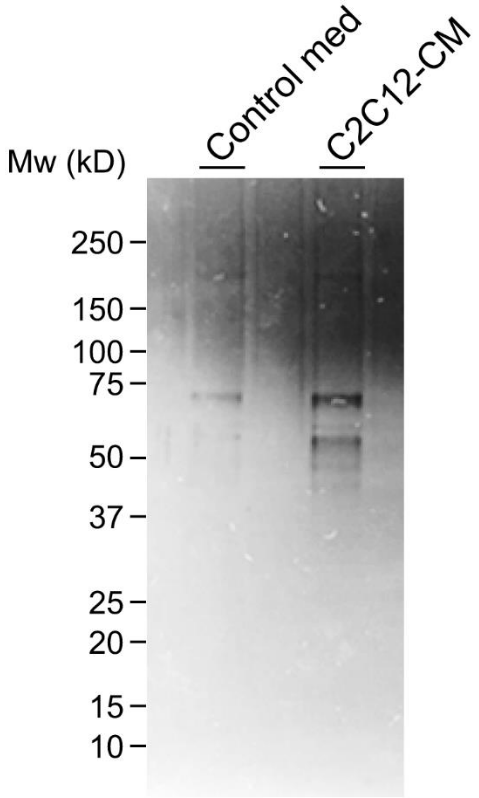

Supplementary Figure 1. Detection of proteins secreted by differentiated C2C12 cells. Equal volumes of control media and C2C12-CM were loaded onto SDS-PAGE. Proteins present in samples were detected using silver staining.

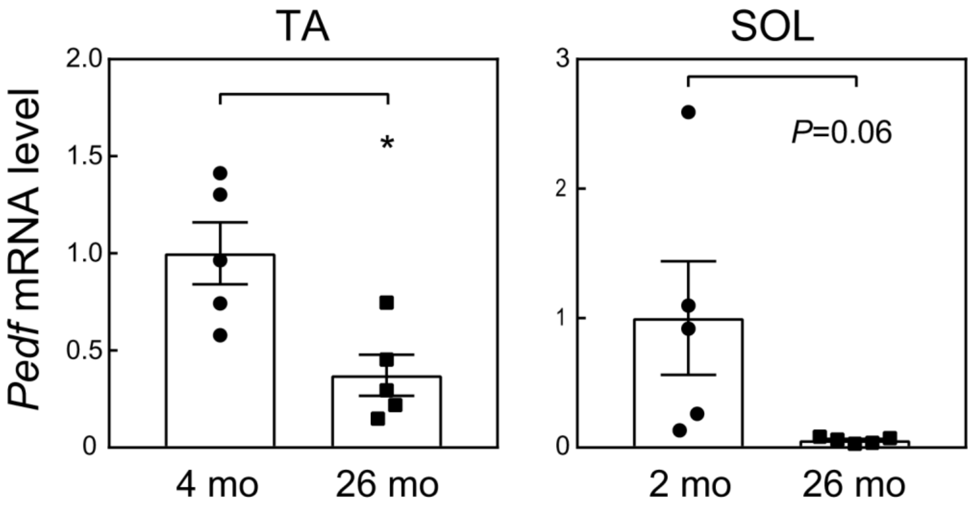

Supplementary Figure 2. *Pedf* expression is reduced in the skeletal muscle of aged animals. Total RNA was isolated from skeletal muscles (TA and SOL), and the expression of *Pedf* was analyzed by real-time PCR. Values were normalized to *Gapdh* in each sample. Values represent means ± SEM. Data were analyzed by the Student's *t*-test (h). *P <0.05.

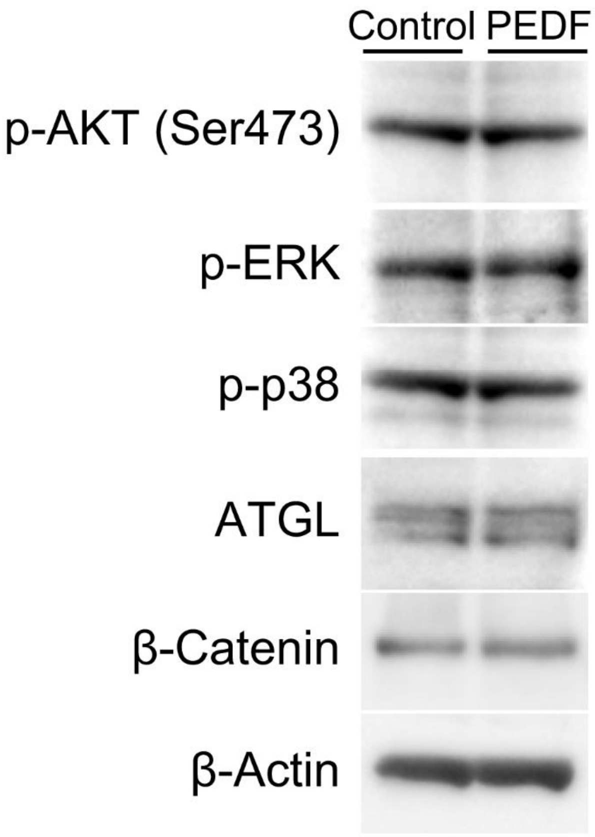

Supplementary Figure 3. Effects of PEDF on signaling molecules. MEFs were stimulated with PEDF for 24 hours. The phosphorylation or expression of the indicated proteins was analyzed by immunoblotting. β-Actin was used as a loading control.

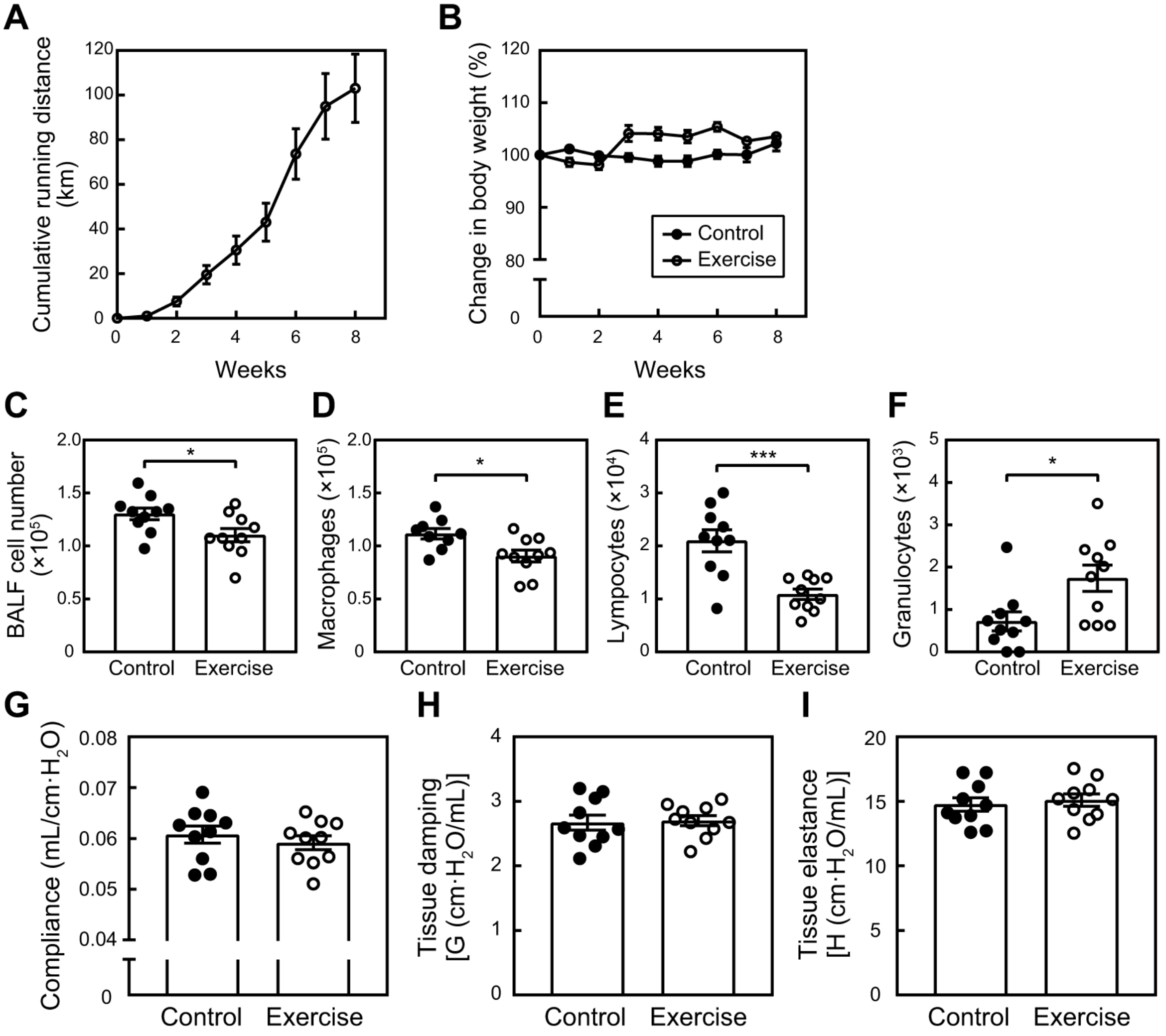

Supplementary Figure 4. Total running distance during voluntary exercise and its effects on body weight and lung parameters. (**A**) The cumulative running distance of mice in Figure 3. (**B**) Changes in body weights during voluntary exercise. (**C**) The number of total BALF cells was counted in each mouse. (**D**–**F**) BALF cells were analyzed by modified Giemsa staining. The number of macrophages (**D**), lymphocytes (**E**), and granulocytes (**F**) in each BALF sample was counted. (**G**–**I**) Mice were subjected to pulmonary function tests. Compliance (**G**), tissue damping (**H**), and tissue elastance (**I**) are shown. Values represent means ± SEM. Data were analyzed by the Student's *t*-test. *P <0.05 and ***P <0.001.

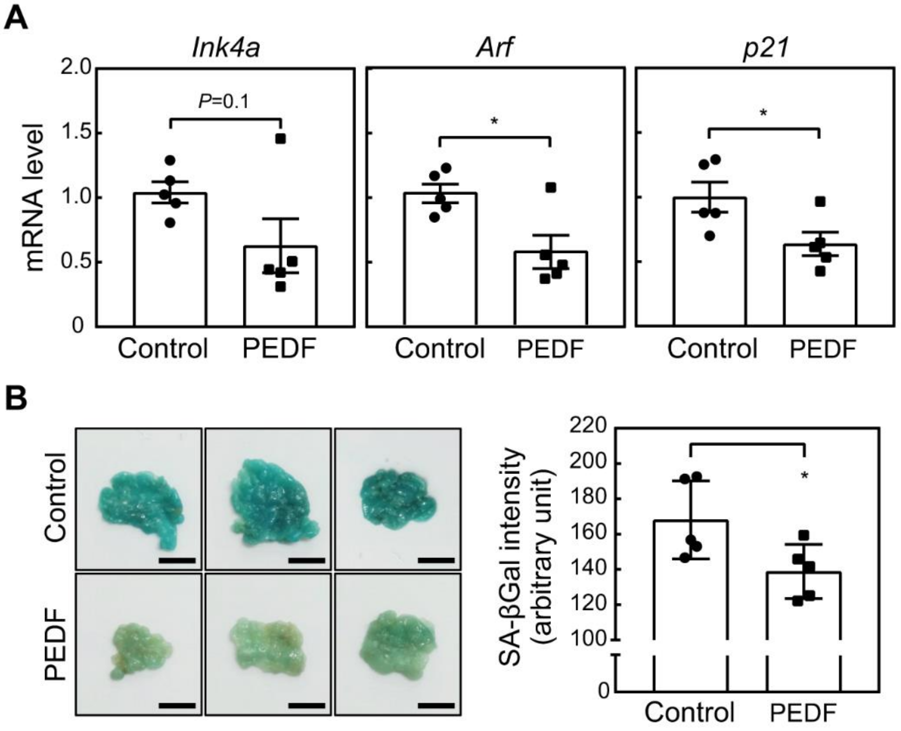

Supplementary Figure 5. PEDF suppresses cellular senescence in adipose tissue. (**A**) The expression of *Ink4a*, *Arf*, and *p21* in perigonadal adipose tissues was analyzed by real-time PCR. Values were normalized to *Gapdh* in each sample. (**B**) *Left*, Fat tissues were stained for SA-β-gal. Representative images are shown. Scale bar, 5 mm. *Right*, SA-β-gal intensity was analyzed in each sample. Values represent means ± SEM. Data were analyzed by the Student's *t*-test. *P <0.05.

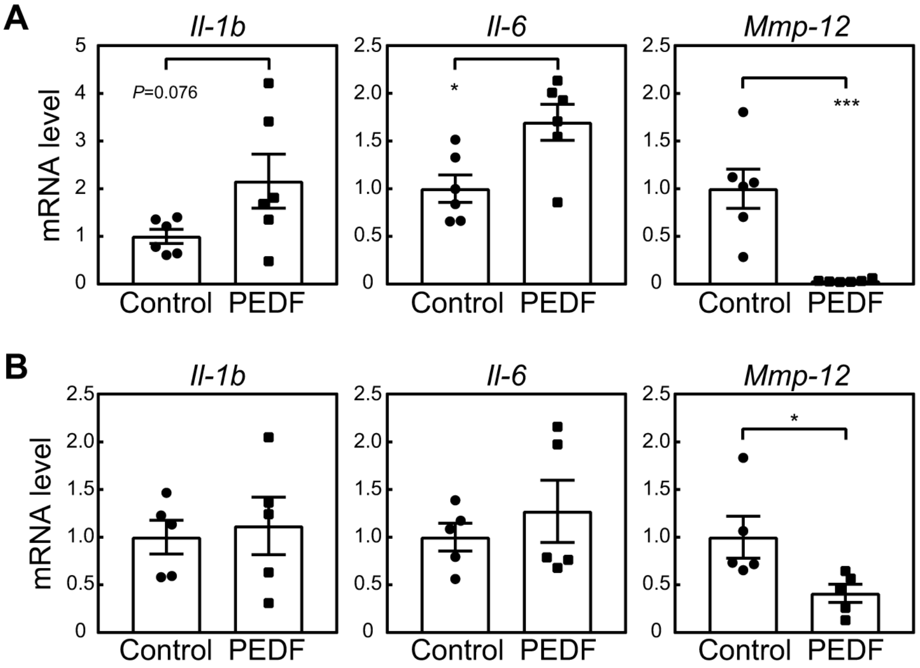

Supplementary Figure 6. Effects of PEDF on the expression of SASP-related factors. Total RNA was isolated from the lung (**A**) and adipose tissues (**B**) of mice in Figure 4, and the expression of the indicated genes was analyzed by real-time PCR. mRNA levels were normalized to *Gapdh* in each sample. Values represent means ± SEM. Data were analyzed by the Student's *t*-test. *P <0.05 and ***P <0.001.

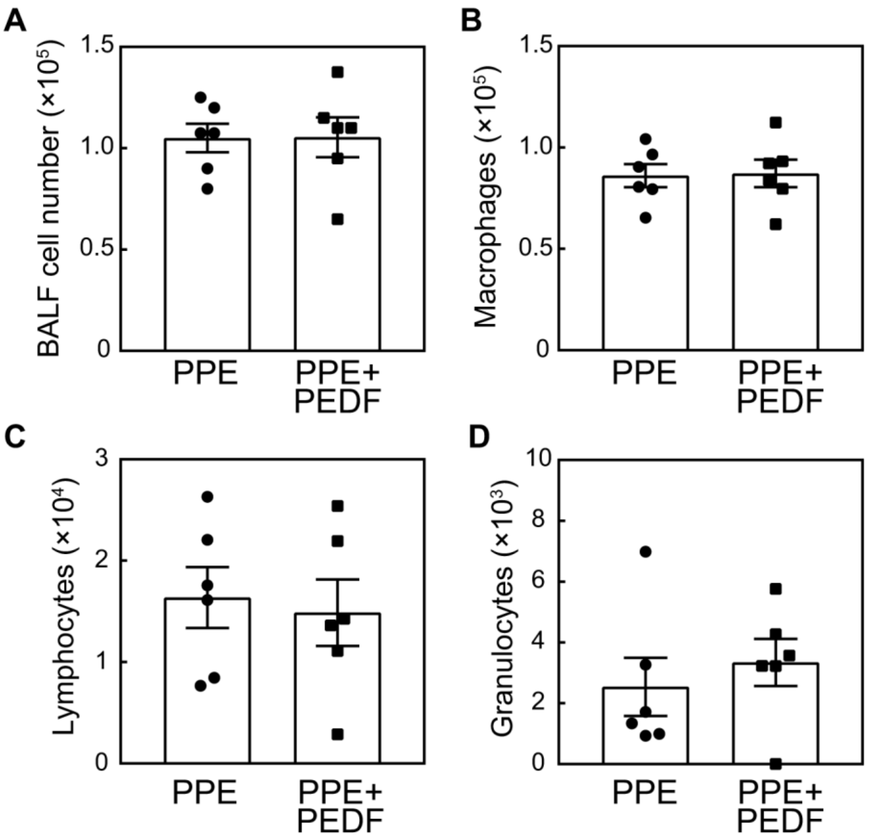

Supplementary Figure 7. PEDF does not affect BALF cells. (**A**) BALF was prepared from mice in Figure 5, and the total cell number in BALF was counted. (**B**–**D**) BALF cells were analyzed by modified Giemsa staining. The numbers of macrophages (**B**), lymphocytes (**C**), and granulocytes (**D**) in each BALF sample were counted. Values represent means ± SEM. Data were analyzed by the Student's *t*-test.

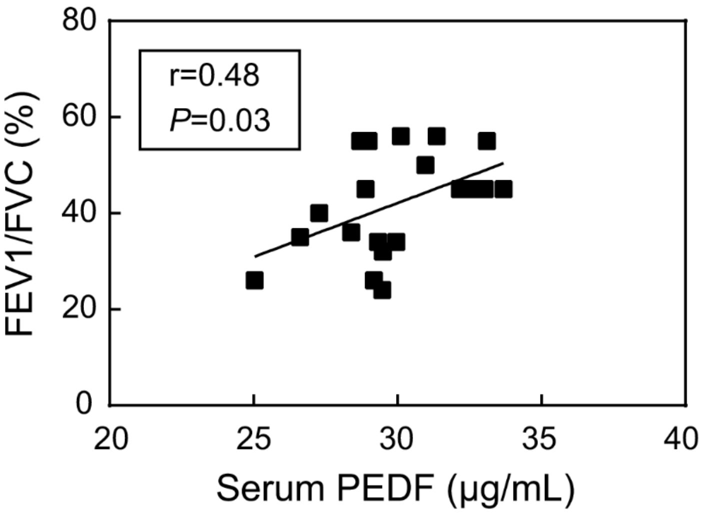

Supplementary Figure 8. Serum PEDF levels correlate with pulmonary function in patients with COPD. Detailed patient information is presented in Table S2. Pearson's correlation and a simple linear regression between serum PEDF levels and FEV1/FVC in patients with COPD (n = 20).
